# Cardioprotective cytokine interleukin‐33 is up‐regulated by statins in human cardiac tissue

**DOI:** 10.1111/jcmm.13891

**Published:** 2018-09-14

**Authors:** Richard Pentz, Christoph Kaun, Barbara Thaler, Stefan Stojkovic, Max Lenz, Konstantin A. Krychtiuk, Andreas Zuckermann, Kurt Huber, Johann Wojta, Philipp J. Hohensinner, Svitlana Demyanets

**Affiliations:** ^1^ Department of Internal Medicine II Division of Cardiology Medical University of Vienna Vienna Austria; ^2^ Department of Surgery Medical University of Vienna Vienna Austria; ^3^ 3^rd^ Medical Department, Cardiology and Intensive Care Medicine Wilhelminen Hospital Vienna Austria; ^4^ Medical Faculty Sigmund Freud Private University Vienna Austria; ^5^ Ludwig Boltzmann Cluster for Cardiovascular Research Vienna Austria; ^6^ Core Facilities Medical University of Vienna Vienna Austria; ^7^ Department of Laboratory Medicine Medical University of Vienna Vienna Austria

**Keywords:** bisphosphonates, cardiac cells, interleukin‐33, pleiotropic, statins

## Abstract

Interleukin (IL)‐33 is a member of the IL‐1 family and is able to act cardioprotective. The aim of this study was to investigate the regulation of IL‐33 by 3‐hydroxy‐3‐methylglutaryl‐coenzyme‐A (HMG‐CoA) reductase inhibitors (statins) and bisphosphonates (BPs) in human cardiac tissue. The lipophilic fluvastatin, simvastatin, atorvastatin, and lovastatin as well as the nitrogenous BPs alendronate and ibandronate, but not hydrophilic pravastatin increased IL‐33 mRNA and intracellular IL‐33 protein levels in both human adult cardiac myocytes (HACM) and fibroblasts (HACF). Additionally, fluvastatin reduced soluble ST2 secretion from HACM. IL‐33 was also up‐regulated by the general inhibitor of prenylation perillic acid, a RhoA kinase inhibitor Y‐27632, and by latrunculin B, but statin‐induced IL‐33 expression was inhibited by mevalonate, geranylgeranyl pyrophosphate (GGPP) and RhoA activator U‐46619. The IL‐33 promoter was 2.3‐fold more accessible in statin‐treated HACM compared to untreated cells (*P* = 0.037). In explanted hearts of statin‐treated patients IL‐33 protein was up‐regulated as compared with the hearts of non‐statin‐treated patients (*P* = 0.048). As IL‐33 was previously shown to exert cardioprotective effects, one could speculate that such up‐regulation of IL‐33 expression in human cardiac cells, which might happen mainly through protein geranylgeranylation, could be a novel mechanism contributing to known cardioprotective effects of statins and BPs.

## 1 INTRODUCTION

HMG‐CoA reductase inhibitors or statins are used for primary and secondary prevention of atherosclerosis‐associated cardiovascular events.^1^ Besides their lipid‐modifying properties, a variety of pleiotropic effects of statins has been discovered: statins function as anti‐inflammatory, anti‐thrombotic and anti‐oxidant agents.^2^, ^3^, ^4^, ^5^

Cardiac tissue remodelling is an important factor in the progression of cardiovascular diseases. Statins are considered to positively affect cardiac remodelling by attenuation of myocardial hypertrophy.^2^, ^6^ Previous investigations from our group revealed modulating effects of statins on the plasminogen activator/plasmin system,^7^ apoptosis,^8^ and inflammation‐triggered B‐type natriuretic peptide 9 in human cardiac cells. These mechanisms are implicated in the organisation of cardiac tissue and, as a consequence, in myocardial hypertrophy.

BPs represent another group of drugs targeting the mevalonate (MVA) pathway and are used for the treatment of osteoporosis and other diseases involving bone resorption.^10^ BPs also exert beneficial functions in the cardiovascular system: they attenuate cardiac hypertrophy as well as fibrosis and improve endothelial function in hypertensive rats and mice, and cause regression of atherosclerosis.^11^, ^12^, ^13^, ^14^ However, the mechanisms underlying the pleiotropic effects of statins and BPs in myocardial tissue remodelling are not yet fully clarified.

IL‐33 belongs to the IL‐1 family of cytokines.^15^ Depending on the experimental model and the pathophysiological context IL‐33 can act either pro‐ or anti‐inflammatory.^3^, ^16^, ^17^, ^18^, ^19^, ^20^ In the heart, IL‐33 is believed to be cardioprotective as it was shown to possess anti‐hypertrophic and anti‐apoptotic properties in animal models of pressure overload or myocardial infarction, respectively.^21^, ^22^, ^23^, ^24^

In this study, we aimed to explore if statins and BPs can influence the expression of IL‐33 in human adult cardiac myocytes and fibroblasts, and to reveal the responsible mechanisms. Additionally, we compared IL‐33 expression in explanted hearts from statin‐treated versus non‐statin‐treated patients with end‐stage HF undergoing heart transplantation.

## 2 MATERIALS AND METHODS

### 2.1 Reagents

Stock solutions of atorvastatin (kindly provided by Pfizer, Sandwich, UK), fluvastatin (kindly provided by Novartis, Basel, Switzerland), pravastatin (kindly provided by Bristol‐Myers‐Squibb, Paris, France), lovastatin and simvastatin (kindly provided by Merck Sharpe and Dome, Ballydine, Ireland) were prepared as described.^25^ Such stocks were aliquoted and stored at −80°C. Alendronate, ibandronate, MVA, GGPP, FPP, squalene, coenzyme Q10, latrunculin B, and perillic acid were purchased from Sigma (St. Louis, MO, USA). Y‐27632 and U‐46619 were purchased from Cayman Chemical Company (Ann Arbor, MI, USA).

### 2.2 Cell culture

Primary HACM and HACF were isolated from myocardial tissue obtained from explanted recipients’ hearts after heart transplantation and cultivated as described by our group.^8^, ^26^, ^27^ For this study, cells were isolated from the hearts of 10 different donors (eight persons >18 years old and two children, mean age 40.5 ± 20.8; six males and four females) suffering from different pathologies (three patients with ischaemic cardiomyopathy, five patients with dilated cardiomyopathy (in one case perinatal), one patient with constrictive pericarditis, and one child with an atrioventricular septal defect).

HACM were isolated from ventricular tissue and HACF from the papillary muscle. For HACM isolation, ventricular tissue was grinded into very small fragments in PBS (pH 7.4) without enzymatic digestion. Red blood cells were removed by rinsing with PBS twice and debris was removed by passing through 40 mm cell strainer (Gibco‐Life Technologies, Paisley, UK). The filtrated suspension was centrifuged at 335 *g* for 10 minutes. Next, the pellet was washed twice with PBS and resuspended in Dulbecco's Minimal Essential Medium (DMEM) supplemented with 10% fetal calf serum (FCS), 100 U/mL penicillin and 100 mg/mL streptomycin (all Cambrex, East Rutherford, NJ, USA). Cells were seeded into a Petri‐dish and incubated for 60 minutes at 37°C in a humidified atmosphere of 5% CO_2_:95% air in order to separate myocytes from fibroblasts by pre‐plating. Afterwards the non‐attached cells were removed, centrifuged, and washed twice with PBS. The cell pellet was resolved in DMEM supplemented with 10% FCS, 100 U/mL penicillin, 100 mg/mL streptomycin, 10 mg/mL transferrin (Sigma), and 10 mg/mL insulin (Sigma). HACM were seeded at the density of around 1 × 10^4^ cells/cm^2^ into fibronectin‐coated (Roche, Basel, Switzerland) culture flasks. Furthermore, cells were cultured at 37°C in a humidified atmosphere of 5% CO_2_:95% air. Only cultures in which >95% of the cells stained positive for cardiac myocyte markers (troponin I [Santa Cruz, Santa Cruz, CA, USA], tropomyosin (Sigma), cardiotin [Chemicon, Temcula, CA, USA] and myocardial muscle actin [Dako, Glostrup, Denmark]) were used in this study. In these cultures contamination with smooth muscle cells, endothelial cells, and fibroblasts as judged by staining for smooth muscle actin (Dako), vWF (Dako) and fibroblast specific antigens (antibody 1B10 and antibody 5B5, both from Dako) was <2%.

For HACF isolation, the explant technique was used. Pieces of myocardial tissue were covered with Medium199 (M199, Sigma) supplemented with 10% FCS, 100 U/mL penicillin and 100 mg/mL streptomycin in a Petri dish. After the explants became adherent, which took 3‐5 days, the Petri dish was filled with fresh M199 supplemented with FBS and antibiotics. HACF growing out from the explants were maintained to confluence and cultured at 37°C in a humidified atmosphere of 5% CO_2_:95% air. >95% of these cells stained positive for fibroblast specific antigen. HACF did neither stain for the cardiac myocyte markers troponin I, tropomyosin, cardiotin, and myocardial muscle actin, nor for the endothelial marker vWF nor for smooth muscle actin ruling out contamination with myocytes, smooth muscle cells, and endothelial cells, respectively. Cells were further cultured in minimum essential medium 199 (M199, Sigma) containing 20% fetal calf serum (FCS), 100 U/mL penicillin, 100 U/mL streptomycin, 0.25 μg/mL fungizone, and 2 mmol L^−1^ L‐glutamine (all Cambrex, East Rutherford, NJ, USA) at 37°C in a humidified atmosphere of 5% CO_2_:95% air. Cells used in this study were between passage 2 and 5. The study has been reviewed and approved by the Ethic Committee of the Medical University of Vienna, Austria.

### 2.3 Treatment of the cells

HACM and HACF were incubated in M199 containing 0.1% bovine serum albumin (BSA; Sigma) for 24 h prior to treatment with the respective substance. Thereafter, the medium was replaced with fresh M199 containing 0.1% BSA, and the cells were treated with different statins (atorvastatin, fluvastatin, lovastatin, simvastatin, or pravastatin) at concentrations from 0.5 to 5 μmol L^−1^ or BPs alendronate or ibandronate at concentrations from 1 nmol L^−1^ to 1 μmol L^−1^ for time periods up to 72 h. In additional experiments HACM and HACF were treated with fluvastatin, ibandronate, or alendronate in the absence or presence of MVA (100 μmol L^−1^), GGPP (10 μmol L^−1^), FPP (10 nm–1 μmol L^−1^), squalene (10 nm–1 μmol L^−1^), coenzyme Q10 (10 nm–1 μmol L^−1^), or U‐46619 (10 nmol L^−1^) for 48 h. Moreover, human cardiac cells were treated with Y‐27632 at 5 μmol L^−1^ or latrunculin B from 10 nmol L^−1^ to 1 μmol L^−1^ for 48 h. The concentrations of the substances used in this study were in the same range as concentrations used in numerous other tissue culture studies, including ours.^3^, ^7^, ^8^, ^9^, ^25^, ^28^, ^29^

### 2.4 Human tissue

Human heart tissue was obtained from the left ventricle of explanted hearts from 10 patients (mean age 53.5 ± 9.3; nine males; five with statin treatment) undergoing heart transplantation for end‐stage HF. Specimens were paraffin‐embedded and cut into 5 μm sections for further analysis. All human material was obtained and processed according to the recommendations of the hospital's Ethics Committee.

### 2.5 Total RNA purification and cDNA preparation

Cells were treated as described, supernatants were removed and total cellular RNA was isolated using High Pure RNA Isolation Kit (Roche, Basel, Switzerland) according to the manufacturer's instructions. Reverse transcription was performed using Transcriptor First Strand cDNA Synthesis Kit (Roche).

### 2.6 RealTime‐PCR

RealTime‐PCR was performed using LightCycler^®^ TaqMan^®^ Master (Roche) according to the manufacturer's instructions. Primers were designed using the Roche UniversalProbeLibrary Assay Design Centre (http://www.universalprobelibrary.com/): glyceraldehyde‐3‐phosphate dehydrogenase (GAPDH) (forward primer: 5′‐agccacatcgctcagacac‐3′, reverse primer: 5′‐gcccaatacgaccaaatcc‐3′, UPL probe #60; Amplicon Size [bp] 66) – IL‐33 (forward primer: 5′‐agcaaagtggaagaacacagc‐3′, reverse primer: 5′‐cttctttggccttctgttgg‐3′, UPL probe #33, Amplicon Size [bp] 74) – sST2 (forward primer: 5′‐gggagagatatgctacctggag‐3′, reverse primer: 5′‐ cgcctgctctttcgtatgtt‐3′, UPL probe #86, Amplicon Size [bp] 68) – ST2L (forward primer: 5′‐ gaaatacctgagactgggtgatttat 3′, reverse primer: 5′‐gaagtgcctgcctttgctt‐3′, UPL probe #29, Amplicon Size [bp] 149). The amplification conditions consisted of an initial incubation at 95°C for 10 minutes, followed by 45 cycles of 95°C for 10 seconds, 63°C for 20 seconds and 72°C for 6 seconds and a final cooling to 4°C. Data were analyzed using LightCycler Software Version 3.5 (Roche).

### 2.7 Promoter accessibility assay

Chromatin accessibility was analysed using a commercially available Chromatin Accessibility Assay Kit (Abcam, Cat.Nr. ab185901) as described.^30^ In short, HACM were treated with fluvastatin at 5 μmol L^−1^ or alendronate at 10 nmol L^−1^ for 24 h followed by cell lysis and chromatin extraction. Chromatin was thereafter digested using a nuclease mix. Following a DNA cleanup, samples were analysed by qPCR using two IL‐33 promoter specific primer (forward primer 1: 5′‐gcagtgcagtgggttgttta‐3′, reverse primer 1: 5′‐agaacaattcattacttgcgtgtt‐3′, UPL probe #9; Amplicon Size [bp] 60; forward primer 2: 5′‐cttcaggggcaataacatgc‐3′, reverse primer 2: 5′‐cctcaggcagttctgtcaaa‐3′, UPL probe #6; Amplicon Size 86 [bp]). Results were calculated by fold enrichment using a ratio of amplification efficiency of nuclease‐treated samples over that of untreated nuclease‐free control samples as suggested in the protocol.

### 2.8 Protein detection

Cells were lysed with PBS containing 0.1% Triton X‐100 (Sigma). IL‐33 protein in cell lysates and sST2 protein in cell culture supernatants were measured by a specific enzyme‐linked immunosorbent assay (ELISA) (both R&D Systems, Minneapolis, MN, USA), as described by us previously.^27^

### 2.9 Immunofluorescence analysis of IL‐33 in explanted hearts

Paraffin‐embedded sections were deparaffinised and then boiled in target retrieval solution (DAKO North America, Inc, CA) for 10 minutes. Staining with either mouse monoclonal anti‐IL‐33 (Alexis Biochemicals, Enzo Life Sciences AG, Lausen, Switzerland, dilution 1:200) or with rabbit polyclonal anti‐Troponin I (abcam, Cambridge, UK, dilution 1:100) antibodies was performed as described.^27^ The IL‐33 fraction of the stained myocardial tissue was quantified using ImageJ software. Mean fluorescence intensity (MFI) was compared between patients with or without statin treatment in order to determine the myocardial IL‐33 content.

### 2.10 Statistics

Values are expressed as mean ± SD. Data were compared by two‐tailed student's *t* test or by ANOVA. Values of *P *≤ 0.05 were considered significant. Each experiment was performed at least three times with cells obtained from different donors. Representative experiments are shown if not indicated otherwise.

## 3 RESULTS

### 3.1 Statins induce IL‐33 and reduce sST2 in human cardiac cells

**Figure 1:**
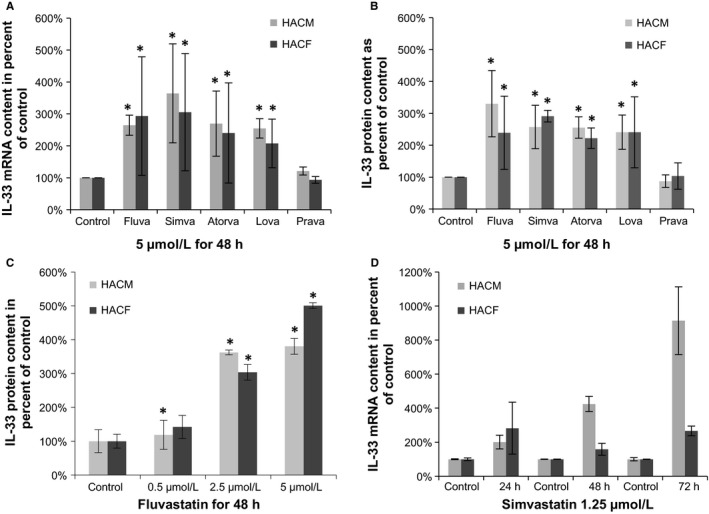
HMG‐CoA reductase inhibitors up‐regulate IL‐33 in HACM and HACF dose‐ and time‐dependently. A and B, HACM and HACF were incubated for 48 h in the absence (control) or presence of fluvastatin (Fluva), simvastatin (Simva), atorvastatin (Atorva), lovastatin (Lova), or pravastatin (Prava) at 5 μmol L^−1^ each. C, HACM and HACF were incubated for 48 h in the absence (control) or presence of fluvastatin at 0.5, 2.5, and 5 μmol L^−1^. D, HACM and HACF were incubated for 24, 48, or 72 h in the absence (control) or presence of simvastatin at 1.25 μmol L^−1^. Values for IL‐33/GAPDH mRNA (A, D) and IL‐33 protein (B, C) are given as % of control, which was set as 100%. A and B show mean ± SD from three different donors, each treated in triplicates (the internal replicates were not included in the analysis), *P ≤ 0.05 as compared to respective control

Treatment of HACM and HACF with the lipophilic fluvastatin, simvastatin, atorvastatin, and lovastatin at 5 μmol L^−1^ for 48 h led to a significant increase in IL‐33 expression on mRNA (Figure 1A) and protein levels (Figure 1B). Hydrophilic pravastatin did not change IL‐33 expression levels (Figure 1A,B).

This stimulatory effect on IL‐33 expression was reproducible in cells derived from different donors’ hearts when the cells were treated with fluvastatin at 5 μmol L^−1^ for 48 h (Table 1). IL‐33 expression was up‐regulated from 1.7‐ to 3.8‐fold in HACM (n = 6 donors) and from 1.5‐ to 3.2‐fold in HACF (n = 4 donors). In a series of experiments where the cells were treated with different concentrations of statins for different time points, we observed that the induction of IL‐33 by statins was concentration‐dependent in both cell types (Figure 1C) and time‐dependent in HACM (Figure 1D). Fluvastatin was effective at 5 μmol L^−1^ and 2.5 μmol L^−1^ in both HACM and HACF, and even at 0.5 μmol L^−1^ in HACM (Figure 1C). Treatment with simvastatin at 2.5 μmol L^−1^ for 24 h, 48 h, and 72 h induced a 3.2‐, 8.2‐, and 17.4‐fold increase of IL‐33 mRNA levels in HACM, and a 2.7‐, 1.5‐, and 2.8‐fold increase of IL‐33 mRNA levels in HACF, respectively (Figure 1D).

**Table 1:** Effect of fluvastatin on intracellular IL‐33 protein in HACM and HACF isolated from different donors

	HACM	HACF
Donor 1	3.3	
Donor 2	2.2	
Donor 3	1.7	
Donor 4	3.8	
Donor 5	3.2	
Donor 6	2.5	
Donor 7		1.5
Donor 8		1.8
Donor 9		3.2
Donor 10		2.9

HACM and HACF isolated from different donors were incubated for 48 h in the absence or presence of fluvastatin at a concentration of 5 μmol L^−1^. Cells were permeabilised with PBS containing 0.1% Triton X‐100, and IL‐33 protein in cell lysates was determined as described in “Materials and methods”. Values are given in x‐fold of control, which was set as 1.0.

sST2 protein was detectable in cell culture supernatants of HACM in three from five tested donors and its secretion was reduced by fluvastatin at 5 μmol L^−1^ to 15% ± 2% after 24 h and to 29% ± 19% after 48 h of treatment compared to the respective controls (both *P* < 0.05). Fluvastatin also showed a trend towards down‐regulation of mRNA specific for sST2 in HACM in five donors tested (to 85% ± 24% after 24 h and to 62% ± 38% after 48 h as compared to the respective controls), but had no influence on the levels of ST2L mRNA (data not shown).

### 3.2 Statin‐induced IL‐33 expression is inhibited by the components of the MVA pathway but not by its non‐prenyl end‐products or FPP

**Figure 2:**
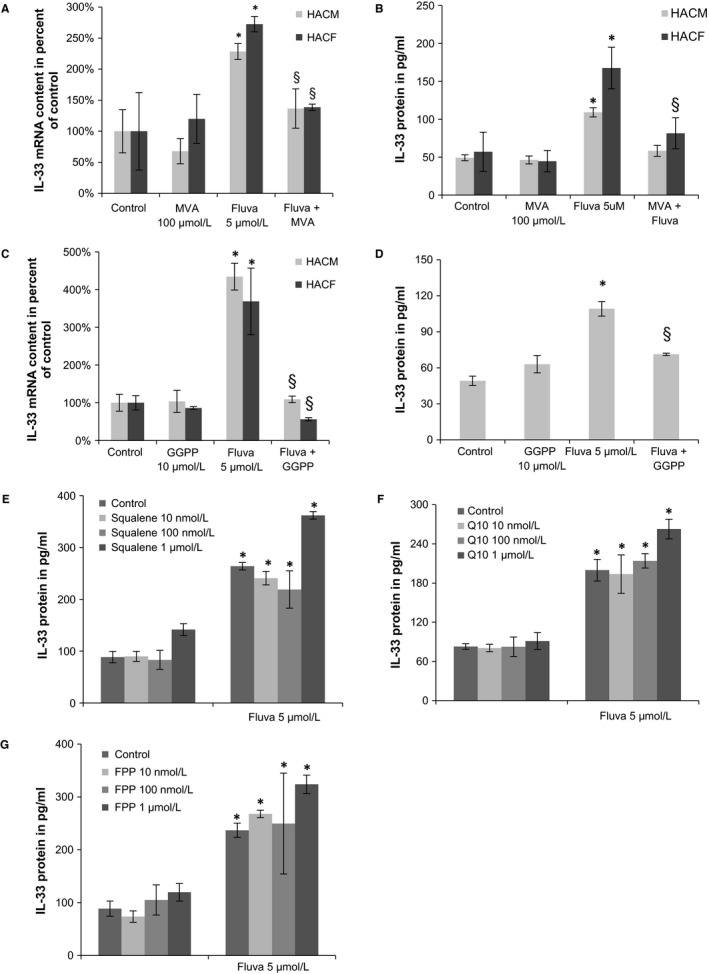
Statin‐induced IL‐33 is inhibited by mevalonate and GGPP, but not by squalene, coenzyme Q10, or FPP in human cardiac cells. A and B, HACM and HACF were incubated for 48 h in the absence (control) or presence of mevalonate (MVA) alone (100 μmol L^−1^), fluvastatin (Fluva) alone (5 μmol L^−1^), or a combination. C, HACM and HACF or D, HACM were incubated for 48 h in the absence (control) or presence of GGPP alone (10 μmol L^−1^), fluvastatin (Fluva) alone (5 μmol L^−1^), or a combination. E, HACM were incubated for 48 h in the absence (control) or presence of squalene alone (10 nmol L^−1^, 100 nmol L^−1^, 1 μmol L^−1^), fluvastatin (Fluva) alone (5 μmol L^−1^), or a combination. F, HACM were incubated for 48 h in the absence (control) or presence of coenzyme Q10 (Q10) alone (10 nmol L^−1^, 100 nmol L^−1^, 1 μmol L^−1^), fluvastatin (Fluva) alone (5 μmol L^−1^), or a combination. G, HACM were incubated for 48 h in the absence (control) or presence of FPP alone (10 nmol L^−1^, 100 nmol L^−1^, 1 μmol L^−1^), fluvastatin (Fluva) alone (5 μmol L^−1^), or a combination. Values for IL‐33/GAPDH mRNA are given as % of control, which was set as 100% (A, C). Values for IL‐33 protein are shown in pg/mL (B, D‐G). *P ≤ 0.05 as compared to respective control; §*P* ≤ 0.05 as compared to fluvastatin‐treated cells

To assess whether the observed effect of statins depends on their blockage of the MVA pathway and to further discriminate, which of the different branches of this pathway are important in this context, we co‐incubated human cardiac cells with fluvastatin and different components or end‐products of the MVA pathway. Fluvastatin‐induced IL‐33 expression in HACM and HACF was inhibited by addition of MVA at 100 μmol L^−1^ (Figure 2A,B) or GGPP at 10 μmol L^−1^ (Figure 2C,D), both of which are metabolites downstream of HMG‐CoA reductase. However, addition of squalene (Figure 2E) or coenzyme Q10 (Figure 2F), two end‐products of the MVA pathway, did not reverse the effect of fluvastatin in human cardiac cells at any concentration used (10 nmol L^−1^, 100 nmol L^−1^, or 1 μmol L^−1^ each). Also addition of FPP, an isoprenoid intermediate of the cholesterol pathway, at different concentrations between 10 nmol L^−1^ and 1 μmol L^−1^ did not abolish fluvastatin‐induced IL‐33 protein increase in HACM (Figure 2G).

To confirm that statin‐induced IL‐33 expression is driven by prevention of protein prenylation, we incubated HACM with perillic acid, which is a general inhibitor of prenylation.^28^ Treatment with perillic acid at 100 μmol L^−1^ for 48 h induced IL‐33 protein 13‐fold in HACM (data not shown).

### 3.3 Bisphosphonates‐induced expression of IL‐33 is reversed by GGPP but not by mevalonate

**Figure 3:**
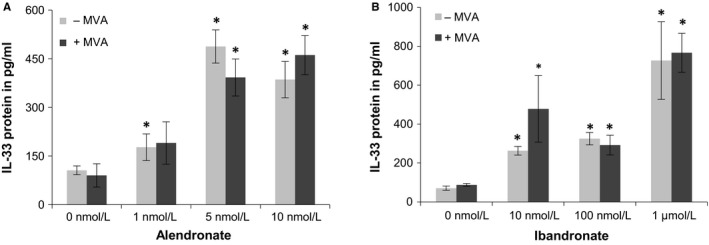
Mevalonate does not reverse bisphosphonates‐induced IL‐33 in human cardiac cells. A, HACM were incubated for 48 h with medium alone (0 nmol L^−1^), MVA (100 μmol L^−1^) alone, alendronate at 1 nmol L^−1^, 5 nmol L^−1^, or 10 nmol L^−1^ alone, or a combination. B, HACM were incubated for 48 h with medium alone (0 nmol L^−1^), MVA (100 μmol L^−1^) alone, ibandronate at 10 nmol L^−1^, 100 nmol L^−1^, or 1 μmol L^−1^ alone, or a combination. Values are given as pg/mL. *P ≤* *0.05 as compared to respective control

Two different BPs, alendronate and ibandronate, significantly up‐regulated IL‐33 protein levels in human cardiac cells (Figure 3). It should be noted that the induction of IL‐33 protein was evident with concentrations from 1 to 10 nmol L^−1^ of alendronate and with 10 nmol L^−1^, 100 nmol L^−1^, and 1 μmol L^−1^ of ibandronate in HACM (Figure 3A,B), whereas in HACF alendronate was effective at a concentration of 10 nmol L^−1^ and ibandronate starting from 100 nmol L^−1^ (data not shown). Thus, we observed cell type‐specific differences in the concentrations of BPs that significantly induced IL‐33 in HACM and HACF. Alendronate at 10 nmol L^−1^ had no effect on the levels of ST2L and sST2 mRNA or secretion of sST2 in HACM from five different donors between 2 h and 48 h of treatment (data not shown). In contrast to fluvastatin, the BPs alendronate and ibandronate induced IL‐33 in HACM even when mevalonate was added at the concentration of 100 μmol L^−1^ (Figure 3A,B). Addition of GGPP had a reversing effect on BPs‐induced IL‐33 expression in both cell types (data not shown).

### 3.4 IL‐33 is increased upon RhoA kinase inhibition and decreased upon RhoA activation

**Figure 4:**
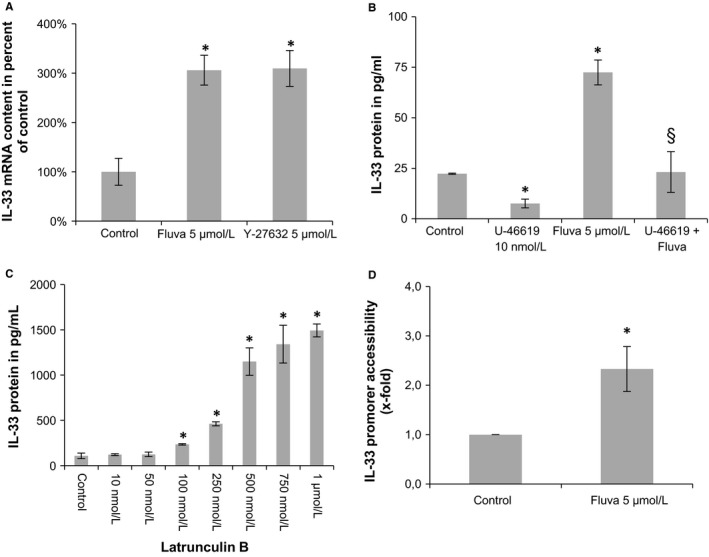
Mechanisms underlying the increase of IL‐33 in human cardiac cells. A, HACM were incubated with medium alone (control), fluvastatin (Fluva, 5 μmol L^−1^), or ROCK inhibitor Y‐27632 (5 μmol L^−1^). B, HACM were incubated for 48 h in the absence (control) or presence of RhoA activator U‐46619 (10 nmol L^−1^) alone, fluvastatin (Fluva, 5 μmol L^−1^) alone or a combination. C, HACM were incubated for 48 h in the absence (control) or presence of latrunculin B at the indicated concentrations. D, HACM were incubated with medium alone (control) or fluvastatin (Fluva, 5 μmol L^−1^) for 24 h. Values for IL‐33/GAPDH mRNA are given as % of control, which was set as 100% (A). Promoter accessibility is given as x‐fold control (D). Values for IL‐33 protein are shown in pg/mL (B, C). *P ≤* *0.05 as compared to respective control; §*P *≤* *0.05 as compared to fluvastatin‐treated cells

One of the most prominent geranylgeranylated protein is RhoA.^31^ We tested if inhibition of ROCK, which is activated by active RhoA, could result in an increase of IL‐33 expression. Indeed, treatment with Y‐27632, a ROCK inhibitor, at 5 μmol L^−1^ increased IL‐33 mRNA levels in HACM to similar extent (approximately 3‐fold) as fluvastatin at the same concentration (Figure 4A). In line with these results, the RhoA activator U‐46619 at 10 nmol L^−1^ significantly decreased both basal and fluvastatin‐induced IL‐33 protein in HACM (Figure 4B). In HACF, there was a trend to the reduction of fluvastatin‐induced IL‐33 protein level with U‐46619 (data not shown).

RhoA activation results in changes in gene expression via activation of serum response factor (SRF).^32^ Treatment of HACM with latrunculin B, which prevents SRF to localise to the nucleus,^33^ at concentrations between 100 nmol L^−1^ and 1 μmol L^−1^ for 48 h led to a significant up to 15‐fold increase of IL‐33 protein levels (Figure 4C).

### 3.5 Epigenetic changes in the promoter region of IL‐33 in statin‐treated human cardiac cells

Epigenetic changes in the promoter region have the potential to change the expression of genes 34 and statins were shown to induce epigenetic changes in cancer cells.^35^ To analyse if statin treatment might have effects on chromatin organisation, we determined the accessibility of the IL‐33 promoter in fluvastatin‐treated HACM. After treatment of HACM with 5 μmol L^−1^ fluvastatin for 24 h, the IL‐33 promoter was on average 2.3‐fold more accessible in statin‐treated compared to untreated cells (*P* = 0.037; Figure 4D). We did not observe a significant change in the IL‐33 promoter region after treatment with alendronate (1.4 fold of control, *P* = 0.6).

### 3.6 Increased IL‐33 expression in myocardial tissue of statin‐treated patients

**Figure 5:**
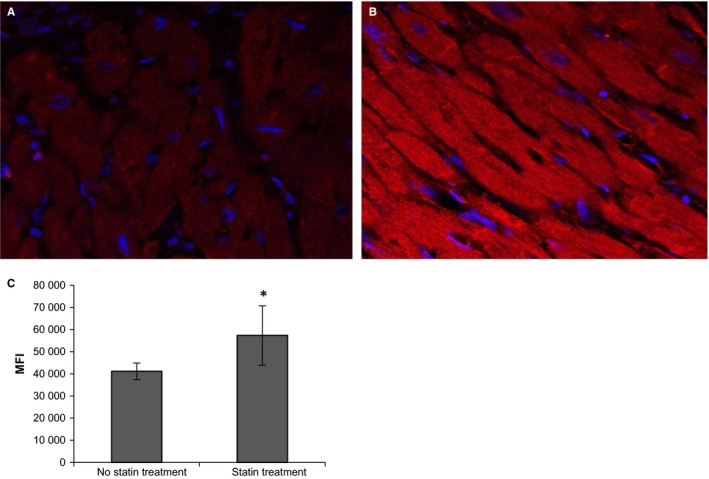
Increased IL‐33 expression in explanted hearts of statin‐treated patients as compared to hearts of non‐statin‐treated patients. A and B, Representative confocal immunofluorescence images of human heart tissue obtained from a non‐statin‐treated patient (A) and a statin‐treated patient (B). Staining for IL‐33 (red) and DAPI (blue). Original magnification × 200. Scale bar = 20 μm. C**,** Quantification of the staining was performed as described in “Materials and Methods”, values are presented as mean fluorescence intensity (MFI) and represent mean values of 5 non‐statin‐treated patients and 5 statin‐treated patients ± SD respectively. *P ≤* *0.05 as compared to non‐statin‐treated patients

In order to support our in vitro data, we additionally examined IL‐33 protein level in myocardial tissue from patients undergoing heart transplantation for end‐stage HF under statin treatment or not treated with statins. As shown in Figure 5B, myocardial tissue from statin‐treated patients express higher level of IL‐33 protein compared with patients without statin treatment (Figure 5A, *P* = 0.048). Quantification of MFI of the staining is shown in Figure 5C.

## 4 DISCUSSION

In our study, we report a novel aspect of action for two widely applied classes of medications: BPs, which are inhibitors of FPP synthase, and HMG‐CoA reductase inhibitors (statins). We found that statins as well as BPs increase the cytokine IL‐33 in human cardiac myocytes and fibroblasts in vitro. Such induction was observed at comparable levels with nitrogen‐containing BPs alendronate and ibandronate and lipophilic statins simvastatin, fluvastatin, lovastatin, and atorvastatin, but not with hydrophilic pravastatin. The induction of intracellular IL‐33 protein as well as mRNA was concentration‐ and time‐dependent, and was reproducible in cells isolated from different donors’ hearts. These results are in accordance with previously published results from our group, which showed that pravastatin had no effect on apoptosis or the expression of components of the plasminogen system and osteoprotegerin in human cardiac, endothelial, or smooth muscle cells in vitro.^7^, ^8^, ^25^, ^36^

In order to support our in vitro findings, we analysed IL‐33 protein by immunofluorescence in human myocardial tissue from explanted hearts of statin‐treated versus non‐statin‐treated patients with end‐stage HF. In accordance with our in vitro data, myocardial tissues from statin‐treated patients express higher amounts of IL‐33 protein as compared to myocardial tissue obtained from non‐statin‐treated patients. However, we have to acknowledge that small sample size (5 statin‐treated vs. 5 not treated failing hearts) is an important limitation of this set of experiments, and besides statin treatment many factors might affect IL‐33 expression in end‐stage HF.

Despite having different primary targets, statins and BPs act as inhibitors of the same MVA pathway.^37^ Though the main clinical indication for statins is to modify blood lipids and BPs are primarily used to suppress bone resorption, ample evidence is available that these two classes of medications exert also other, so‐called pleiotropic, effects.^2^, ^5^, ^6^, ^11^ Previous studies have shown that statins inhibit cardiomyocyte hypertrophy in vitro and in vivo.^6^, ^38^ The mechanisms involved are an antioxidant effect implicating inhibition of Rac1, the inhibition of Rho kinase, p21ras inactivation and a down‐regulation of microRNA‐22.^6^, ^39^ Clinical studies investigating the effects of statin treatment in patients with HF are contradictory. The CORONA and GISSI‐HF trials failed to demonstrate prognostic benefits of statins in patients with HF.^40^, ^41^ On the other hand, the TOPCAT (Treatment of Preserved Cardiac Function Heart Failure with an Aldosterone Antagonist) trial was associated with improved outcome in patients with HF with preserved ejection fraction,^42^ and the CHART‐2 (Chronic Heart Failure Registry and Analysis in the Tohoku District 2) study concluded that higher‐intensity statin use is associated with beneficial outcome in HF patients with ischaemic heart disease.^43^

Interestingly, an overexpression of FFP synthase, the primary target of BPs, induced cardiac hypertrophy and HF in mice.^44^ Furthermore, inhibition of FPP synthase attenuates hypertrophy in neonatal rat cardiomyocytes.^45^ Therefore, both statins and BPs can act as anti‐hypertrophic agents in different experimental models but the exact mechanisms are not clarified yet.

IL‐33 is the most recently identified cytokine of the IL‐1 family.^15^ Intracellular IL‐33 expression was shown in endothelial, epithelial, smooth muscle cells, keratinocytes, and fibroblasts.^15^, ^17^, ^21^, ^27^, ^46^ IL‐33 is released by necrotic cells during damage or by living cells during mechanical strain.^47^, ^48^ We showed previously the intracellular expression of IL‐33 in human cardiac myocytes and fibroblasts, and the release of IL‐33 during necrosis of these cells.^27^ Moreover, the “decoy” receptor for IL‐33, sST2, was shown to have predictive value in patients with coronary artery disease and HF^16^, ^49^, ^50^, ^51^ as well as in critically ill patients.^52^ In our study presented here, we found that fluvastatin is able to reduce secretion of sST2 from human cardiac myocytes. Interestingly, a recent report showed that sST2 levels were attenuated after 12 months of atorvastatin treatment in patients with HIV.^53^ However, rosuvastatin had no effect on the levels of sST2 in patients with HF from the CORONA study though the change in sST2 levels was evaluated after 3 months of rosuvastatin treatment.^54^ In the light of our in vitro data about down‐regulation of sST2 secretion by fluvastatin, it would be warranted to evaluate a possible influence of long‐term statin treatment on sST2 blood levels in clinical studies.

As an inflammatory mediator IL‐33 plays a role in a variety of diseases such as bronchial asthma, inflammatory bowel diseases, and rheumatoid arthritis.^16^ On the other hand, IL‐33 antagonised pro‐hypertrophic stimuli in rat neonatal cardiomyocytes and exhibited an anti‐hypertrophic effect after transverse aortic constriction (TAC) in mice.^21^ Furthermore, IL‐33 protected rat neonatal cardiomyocytes from hypoxia‐induced apoptosis and improved cardiac function in rats after experimental myocardial infarction.^22^ IL‐33 also attenuated ischaemia/reperfusion‐induced myocardial injury in diabetic mice.^23^ Recently, a cardioprotective role of the IL‐33/ST2 system was shown in IL‐33 knockout (IL‐33^‐/‐^) mice subjected to TAC, which exhibited exacerbated left ventricular hypertrophy, aggravated fibrosis, and impaired survival compared to wild‐type littermates after TAC.^24^ Therefore, IL‐33 has been recognised as a cardioprotective cytokine. In this context, it should be emphasised that the effects of statins on IL‐33 expression in human cardiac cells were never studied before. Only one previous study showed that simvastatin induced IL‐33 mRNA expression in human endothelial cells.^55^

We further investigated, which mechanisms are responsible for the increase of IL‐33. It was shown previously that the MVA pathway inhibitors simvastatin and ibandronate affect epigenetic regulation in cancer cells.^35^ We demonstrate here that the promoter region of IL‐33 comprises a more open chromatin structure in fluvastatin‐treated cardiac cells compared to untreated cells as analysed by promoter accessibility. Therefore, we suggest that statin‐treated human adult cardiac myocytes up‐regulate IL‐33 expression via epigenetic changes in the IL‐33 promoter region. Alendronate did not cause significant changes in the IL‐33 promoter region in human cardiac myocytes.

Statins inhibit HMG‐CoA reductase, an enzyme located at the apex of the MVA pathway, whereas second‐ and third‐generation aminobisphosphonates such as alendronic acid and ibandronic acid, inhibit FPP synthase.^37^ The production of FPP and GGPP occurs in a side branch of the MVA pathway and these substances are attached to a variety of proteins as post‐translational modifications known as protein prenylation (protein farnesylation and geranylgeranylation).^56^

We showed that GGPP abolished IL‐33 expression induced by both statins and BPs, whereas only the effect of statins on IL‐33 expression was reduced by MVA supplementation. BPs‐induced IL‐33 induction was not suppressed by MVA, which can be explained by the fact that the BPs’ target FPP synthase is downstream of MVA in the MVA pathway. Additionally, we found that squalene, the precursor of cholesterol, or coenzyme Q10, another end‐product of the MVA pathway, which is responsible for energy production, did not change the effect of statins on IL‐33 expression. This indicates that statin‐induced IL‐33 expression is not driven by lipid depletion or energetic defects but rather caused by prevention of protein prenylation. The finding that perillic acid, a general inhibitor of protein prenylation via inhibition of both geranylgeranyl transferase 1 and Rab geranylgeranyl transferase, also led to an increase of IL‐33 expression in human cardiac myocytes, further supports the notion that the effect of statins and BPs are dependent on the inhibition of this post‐translational modification. FPP could not reverse the effect of statins in our study, which led us to conclude that IL‐33 induction is dependent on geranylgeranylation but not farnesylation.

**Figure 6:**
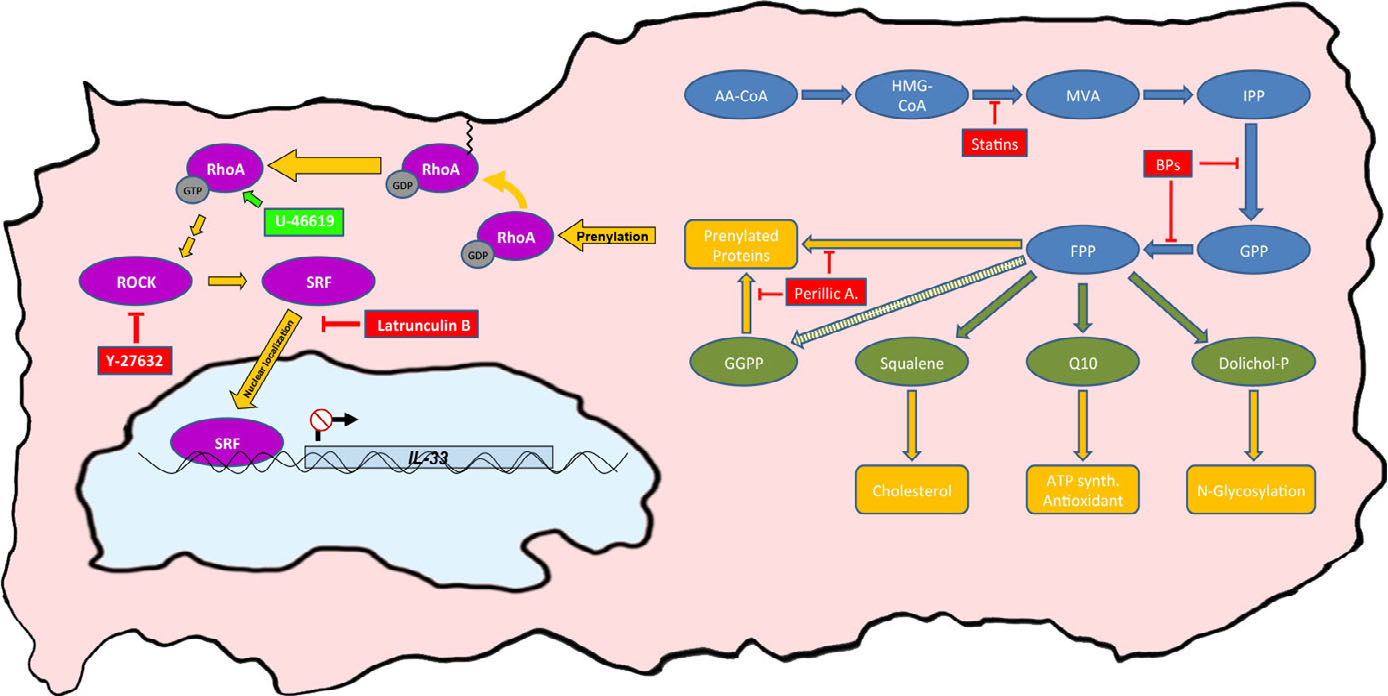
Schematic representation of the downstream targets of the MVA pathway, depicting its various intermediates (blue), end products (green) and functional implications (yellow). Prenylation of RhoA with GGPP anchors RhoA to the plasma membrane, where it can be activated by signalling factors. Active RhoA activates ROCK, which triggers activation and translocation of SRF into the nucleus, where it represses the transcription of IL‐33. Inhibitors used in this study are depicted in red: statins inhibit HMG‐CoA‐reductase; BPs inhibit GPP synthase and FPP synthase; perillic acid inhibits protein prenylation; latrunculin B prevents SRF activation; Y‐27632 is a ROCK inhibitor. The RhoA activator U‐46619 is depicted in green

RhoA is a prominent geranylgeranylated protein, which has also been associated with the diseases of the cardiovascular system and bone.^31^ Statins block the isoprenylation and thus the membrane targeting and functional activation of the Rho family member.^31^ It has been shown that fluvastatin reduces RhoA in the membrane fraction of rat cardiac myocytes.^29^ In spontaneously hypertensive rats simvastatin attenuated RhoA activity and myocyte hypertrophy.^57^ The most important direct target of RhoA is ROCK. We therefore suspected RhoA and subsequent ROCK inactivation of being responsible for the effect of statins and BPs observed by us in human cardiac cells. We confirmed our hypothesis by treating these cells with the ROCK inhibitor Y‐27632. We found that Y‐27632 at 5 μmol L^−1^ exerts a similar induction of IL‐33 in human cardiac cells as fluvastatin at the same concentration. Moreover, U‐46619, a ROCK activator, reduced basal and fluvastatin‐induced IL‐33 levels in our study. RhoA can regulate muscle specific gene expression through activation of SRF.^31^, ^32^ We inhibited nuclear localisation of SRF and thereby its impact on gene expression by latrunculin B and again observed significant and concentration‐dependent increase in IL‐33 levels in human cardiac myocytes. Taken together, our results suggest that statins up‐regulate IL‐33 via impaired geranylgeranylation of RhoA and resulting ROCK inhibition, which entails an inactivation of SRF and its effect on gene expression. An overview of the analysed pathways and results is given in Figure 6.

It should be noted that the concentrations of statins and BPs as well as the components of the MVA pathway and specific inhibitors used in this study were in the same range as concentrations of these substances used in numerous other tissue culture studies.^3^, ^7^, ^8^, ^9^, ^14^, ^25^, ^29^, ^36^

In conclusion, our study showed for the first time that IL‐33 could be an important mediator of the pleiotropic effects of statins and BPs in human cardiac tissue. Considering potential safety issues, the feasibility and possible implications of the therapeutic modulation of IL‐33 signalling, which is known to act cardioprotective,^21^, ^22^, ^23^, ^24^ are currently discussed.^58^ As statins and BPs represent widely used and standardised classes of drugs for primary and secondary prevention of cardiovascular disease, our results might have impact also in the clinical setting.

## CONFLICT OF INTEREST

The authors confirm that there are no conflicts of interests.
